# Stem cell technology for antitumor drug loading and delivery in oncology

**DOI:** 10.32604/or.2023.046497

**Published:** 2024-02-06

**Authors:** FRANCESCO PETRELLA, ENRICO MARIO CASSINA, LIDIA LIBRETTI, EMANUELE PIRONDINI, FEDERICO RAVEGLIA, ANTONIO TUORO

**Affiliations:** Department of Thoracic Surgery, Fondazione IRCCS San Gerardo dei Tintori, Monza, 20900, Italy

**Keywords:** Mesenchymal stromal cell, Drug loading, Drug delivery, Mesothelioma, Melanoma, Glioblastoma, Pancreatic ductal adenocarcinoma, Multiple myeloma

## Abstract

The main aim of antineoplastic treatment is to maximize patient benefit by augmenting the drug accumulation within affected organs and tissues, thus incrementing drug effects and, at the same time, reducing the damage of non-involved tissues to cytotoxic agents. Mesenchymal stromal cells (MSC) represent a group of undifferentiated multipotent cells presenting wide self-renewal features and the capacity to differentiate into an assortment of mesenchymal family cells. During the last year, they have been proposed as natural carriers for the selective release of antitumor drugs to malignant cells, thus optimizing cytotoxic action on cancer cells, while significantly reducing adverse side effects on healthy cells. MSC chemotherapeutic drug loading and delivery is an encouraging new area of cell therapy for several tumors, especially for those with unsatisfactory prognosis and limited treatment options available. Although some experimental models have been successfully developed, phase I clinical studies are needed to confirm this potential application of cell therapy, in particular in the case of primary and secondary lung cancers.

## Introduction

The main aim of antineoplastic chemotherapy is to maximize patient benefit by augmenting the drug accumulation in the target organs and tissues, thus incrementing therapeutic efficacy and, at the same time, reducing the exposure of healthy tissues to cytotoxic agents. Nanomedicines significantly condition the tissue delivery of antineoplastic drugs, thereby significantly decreasing the dose-limiting negative effects while retaining or even enhancing their efficacy [1].

Several methods have been described for selective drug loading and delivery: bacteria, viruses, cells, polymer-based or synthetic lipid carrier systems as well as extracellular vesicles [2]. Mesenchymal stromal cells (MSC) represent a family of undifferentiated multipotent adult cells presenting wide self-renewal features and the capacity to differentiate into an assortment of mesenchymal lineage cells [3,4]. They show wide-ranging anti-inflammatory and immunomodulatory effects on the immune system and-after transplantation-are able to cross-talk with the local microenvironment, stimulating tissue restoring and regeneration. MSC have been extensively employed in the area of regenerative and reparative medicine, both in experimental and clinical settings [5,6]. During the last year, they have been proposed as natural vectors for the targeted release of antineoplastic drugs to malignant cells, thus optimizing the cytotoxic action on cancer cells, while significantly reducing adverse side effects on healthy cells [7].

In this review, we focus on MSC drug loading and delivery as a possible new instrument in daily clinical practice for oncologists, with a dedicated overview of the most commonly used antineoplastic drugs like paclitaxel, gemcitabine, and pemetrexed, in particular in thoracic oncology.

## Mesenchymal Stromal Cells

MSC are multipotent non-differentiated adult cells described as plastic-adherent, fibroblast-like cells presenting extensive self-renewal characteristics and the *in vivo* and *in vitro* ability to differentiate into chondrogenic, osteogenic and adipogenic lineages when properly cultured in dedicated inducing media [8].

MSC show an immune phenotype escaping the host immune system, thus permitting allogenic transplantation without the need for immunosuppression [8]. They are described as major histocompatibility complex II negative cells, lacking costimulatory molecules and having an immune phenotype which permits them to escape the host immune system, thus allowing allogenic transplantation without the need for immunosuppression.

The anti-inflammatory and immunomodulatory properties of MSC have been extensively explored and applied in the gastrointestinal tract, in particular in the case of inflammatory bowel disease and graft versus-host disease. Moreover, it has recently been reported that MSC deriving from Crohn’s patients disclose indoleamine 2,3-dioxygenase mediated immune suppression. After transplantation into host organs and tissues, MSC are able to interconnect with the local microenvironment, thus promoting wound healing and tissue repair by cross-talking with other cell populations within the injured tissues [9]. MSC were initially found in bone marrow but now it is well known that they can be easily isolated from a heterogeneous range of adult and fetal tissues like amniotic fluids and placenta, umbilical cord, periosteum, adipose tissue, dental pulp, tendon, cornea, spleen, thymus, liver, brain and synovial fluid. MSC can additionally differentiate into cells from mesodermal, endodermal and ectodermal lineages such as skeletal myocytes, cardiomyocytes, tenocytes, hepatocytes, endothelial cells, insulin-producing cells, neuronal cells, photoreceptor cells, renal tubular epithelial cells and epidermal and sebaceous duct cells [10]. Interestingly, MSC can scatter to sites of damage and engraft into injured tissues, reacting to cytokines, chemokines and growth factors and producing on-site restorative effects by paracrine secretion of anti-inflammatory molecules and wound-healing factors [11]. For this reason, MSC have been advocated as an experimental treatment for several diseases, by acting via the secretion of paracrine mediators which can reduce loco-regional inflammation, interact with the immune system and stimulate the host repair pathways. In addition, it has been recently observed that MSC are able to produce extracellular vesicles and exosomes, inducing significant therapeutic modifications within the injured host tissues [12].

## Drug Loading and Drug Delivery

New techniques of cell-based delivery of antineoplastic drugs have recently been widely studied to maximize the concentration in neoplastic tissues and minimize adverse side effects in healthy non neoplastic organs and tissues. As MSC are able to migrate to neoplastic tissues and engraft after intravenous injection, they have been recently advocated as a further tool for selective antineoplastic drug loading and delivery [13]. In fact, when exposed to high doses of antineoplastic drugs, MSC disclose intracellulary accumulation of the drug and subsequent delivery without any genetic impairments, thus reducing cancer proliferation [14].

Several techniques for drug loading and delivery have been proposed in the last years, among which immuno-conjugates for selecting cancer-specific antigens, genetically modified stem cells and nanoparticles are the most relevant. With regard to stem cell modifications, glycoengineering protocols to produce exhibition of non-natural azide groups on the external part of MSC-without conditioning their viability or cancer-homing properties-have been described, and nano-engineered MSC were obtained by conditioning human MSC with drug-loaded polymeric nanoparticles [15–18].

Nevertheless, MSC probably represent the ideal option for antineoplastic drug delivery as they promptly comply with culture variables and migrate to neoplastic tissue when injected *in vivo*, presenting intrinsic antitumoral properties. However, it should be clearly emphasized that this technique–at the moment–represents only a preclinical experimental approach since only experiments on laboratory animals or cell lines have been performed, although with encouraging results. An additional property that makes MSC even more attractive, is the ability to cross the blood-brain barrier, thus playing a possible additional role in adult and pediatric central nervous system neoplasms [19,20]. Many theses have been advocated to demonstrate MSC antintumoral activity, ranging from the blocking of proliferation-related signaling pathways to angiogenesis and cell cycle inhibition [21]. On the other hand, some doubts still exist about the potential risk of tumor boost by MSC instead of tumor suppression, thus making the shift from bench to bedside even more difficult [22]. The antineoplastic drugs that are most effectively loaded by MSC are gemcitabine and paclitaxel; in addition, although pemetrexed has disclosed encouraging *in vivo* results for pleural mesothelioma treatment, it is not properly internalized by the cells and, for this reason, cannot at the moment benefit from this approach.

## Drugs that Can Be Delivered by MSC

*Pemetrexed*: this acts by inhibiting folate-dependent enzymes and stopping DNA and RNA replication by nucleobase biosynthesis. The inhibitors of folate metabolism play a crucial role, particularly in the treatment of hematologic and solid tumours.

*Paclitaxel:* this is a plant-derived drug extracted from *Taxus brevifolia* and it is used in a wide range of neoplasms; it mainly acts by conditioning the depolarized/polarized equilibrium.

*Gemcitabine:* this is a nucleoside analogue resulting from the combination of a deoxy-difluorinated D-ribose and a pyrimidine base (cytosine). It acts by inhibiting ribonucleotide reductase and DNA synthesis and has shown antineoplastic activity against many types of solid tumors.

*Other drugs:* during the last few years several other antineoplastic drugs have been studied as potential carriers by MSC; in a preclinical study by Kosaka et coll., concurrent administration of 5-fluorocytosine and MSC bound to cytosine deaminase led to significant survival improvement in rats with gliomas [23]; similarly, Ryu et coll. disclosed that thymidine kinase loaded MSC by herpes simplex I virus improved overall survival in a similar population of mice [24]. Xiao et al. reported that silica nanorattle-doxorubicin compounds can be fixed to MSC in nanoparticulate patches which are able to migrate towards U251 tumor cells both *in vivo* and *in vitro* [25]. Rachakatla et coll. disclosed that neural progenitor cells–embedded with magnetic nanoparticles and distributed to mice affected by melanoma–were able to significantly reduce tumor volume after the application of an alternating magnetic field [26].

## Future Perspectives and Potential Clinical Scenarios

***Pancreatic ductal adenocarcinoma (PDAC):*** this is the most frequent cancer of the pancreas and it is the fourth cause of death due to neoplastic disease in the USA [27]. It shows a strong resistance to radiotherapy and chemotherapeutic approaches, thus resulting in an extremely poor overall 5-year survival of 5% [28]. Standard first-line therapy for locally advanced and metastatic PDAC is a combination of gemcitabine (GCB) and 5-fluorouracil, with a significant clinical response in about 10% of cases, indicating that innovative treatments are urgently required [29]. It has been shown that GCB-loaded MSC are able to incorporate into the cancer mass and release much higher concentrations of drugs than those delivered by intravenous infusion, thus playing as a “Trojan horse” for drug delivery into the tumor. More specifically, MSC might enhance tumor growth by migrating from the bone marrow to blood vessels of pancreatic cancer after the hypoxia-induced secretion of several growth factors; being loaded with an antitumoral agent, they could be integrated into the tumor mass and deliver the drug *in situ* at much higher concentrations than those obtained by intravenous injection [13].

***Glioblastoma multiforme (GBM):*** it represents about 15% of all brain tumors and it is the central nervous system tumor with the worst prognosis. Standard treatment of GBM is represented by surgical resection and postoperative chemo-radiotherapy; however, despite the multimodality approach, it frequently recurs with overall 5-year survival rate of less than 5% and a survival time after diagnosis ranging from 12 to 15 months [30]. In experimental preclinical settings on small animals, MSC have been shown to migrate and engraft into GBM neoplasms xenografts, both in the case of intravenous administration or local intracerebral injection. The interaction between implanted MSC and local tumor microenvironment resulted in longer overall survival, tumor volume decrease as well as tumor cell proliferation and vascularization impairment, thus supporting the hypothesis of a potential contribution of MSC in neuro-oncology [30].

It should be emphasized that different glioblastoma cancer cells disclose contradictory behavior after treatment with adipose derived MSC. In fact, in several cancer lines, adipose derived MSC inhibits cell proliferation [31,32] while in some other tumor cells, MSC enhances cell proliferation thus stimulating invasion and migration [33,34].

***Melanoma:*** This is a malignant tumor arising from melanocytes of the skin or other districts. Surgical excision represents the best therapeutic option for local diseases, while chemotherapy, biologic therapy, immunotherapy and radiotherapy play a pivotal role in metastatic disease, although the 5-year overall survival rate remains poor in this setting. Immunotherapy in melanoma obtained excellent overall survival results, in particular in metastatic patients and boosted immunotherapy research in other solid tumors; however, overall survival data remain globally poor and new therapeutic options are constantly explored. Paclitaxel-loaded MSC disclosed effective lung metastase inhibition in a murine melanoma model, thus representing a further therapeutic option for lung metastases from melanoma [35].

***Multiple myeloma (MM)*** is a tumor that suppresses osteoblastogenesis by bone marrow-derived MSC. The potential contribution of MSC to MM treatment is somewhat controversial because of the unclear results about the MSC property of inhibiting or boosting tumor growth. MSC might be used as carriers for selective delivery of antitumoral drugs into bone marrow. In fact, it has been experimentally shown that paclitaxel-loaded MSC are able to suppress myeloma cell growth by acting on the proliferation cell cycle of human myeloma cell lines [36]. Moreover, it has recently been demonstrated that apoptotic MSC are able to interiorize externally included nanoparticles into apoptotic vesicles with an elevated loading capacity. When nano-bortezomib is encapsulated into apoptotic vesicles, the resulting compound discloses a synergistic combination effect of bortezomib and apoptotic vesicles, thus improving multiple myeloma in a mouse model [37].

***Pleural mesothelioma (PM)*** is a neoplastic disease due to asbestos exposure which mainly affects professionally exposed workers who have worked with asbestos for long periods. At the moment, the standard first-line therapy is a platinum-based doublet including a third-generation antifolate such as raltitrexed or pemetrexed; although immunocheckpoint inhibitors have shown promising results in certain clinical settings, there is no officially approved therapy for second-line approach to PM and its prognosis remains very poor; an alternative therapeutic option is thus urgently needed.

The first experimental approaches disclosed that pemetrexed or raltitrexed was not properly internalized by MSC, thus not representing an effective innovative option for this disease; on the other side, paclitaxel-loaded MSC disclosed a good capacity to suppress MPM cell proliferation, thus representing a possible further option for second line treatment of PM (Table 1). More in-depth, our group recently demonstrated that PMX–which still represents the first line treatment-together with platinum–for PM, might not be internalized and released by MSC and so, supported by our previous results of PTX priming of MSC, we tested the inhibition efficacy of PTX-loaded MSC on biphasic PM cell proliferation. We observed that MSC loaded with PTX disclosed a superb ability to block PM cell proliferation, thus currently representing the best “*in vitro*” combination for MPM treatment.

**Table 1:** MSC key findings for each cancer type

Tumor	MSC administration	MSC role
Pancreatic ductal adenocarcinoma	Gemcitabine loaded MSC	To blend into the cancer mass and release much higher local concentrations
Glioblastoma multiforme	MSC injection (intravenous or intracerebral)	Tumor cell proliferation and vascularization impairment
Melanoma	Paclitaxel-loaded MSC	Lung metastases inhibition in mice melanoma model
Multiple myeloma	Paclitaxel-loaded MSC	To suppress neoplastic cell growth by acting on the proliferation cell cycle of human myeloma cell lines
Pleural mesothelioma	Paclitaxel-loaded MSC	To suppress MPM cell proliferation

## Conclusions

MSC chemotherapeutic drug loading and delivery is an encouraging new area of advanced cell therapy for neoplastic diseases, especially for those with a poor prognosis and limited available treatment options [38–41]. Although some experimental models have been successfully developed [42,43], phase I clinical studies are needed to confirm this potential application of cell therapy [37]. As MSC have been shown to absorb and then discharge several types of drugs (e.g., gemcitabine, paclitaxel, doxorubicin for oncologic purposes), we believe that MSC could boost their basal antineoplastic properties once loaded with chemotherapy agents. Whether this cell therapy approach could represent a new adjuvant treatment to standard existing therapies–including surgery-for some tumors remains to be further investigated by clinical trials with large-scale production in bioreactors, according to the good manufacturing practice requested by the international regulatory agencies.

## Data Availability

Not required for review articles.

## Abbreviations

MSC: Mesenchymal stromal cells
PDAC: Pancreatic ductal adenocarcinoma
GCB: Gemcitabine
GBM: Glioblastoma multiforme
PM: Pleural mesothelioma
MM: Multiple myeloma

## References

[1] Yadav, R., Das, P. P., Sharma, S., Sengupta, S., Kumar, D. et al. (2023). Recent advancement of nanomedicine-based targeted delivery for cervical cancer treatment. Medical Oncology*,* 40 *(*12*),* 347; 37930458 10.1007/s12032-023-02195-3

[2] Song, X., Zhang, Y., Tang, Z., Du, L. (2024). Advantages of nanocarriers for basic research in the field of traumatic brain injury. Neural Regeneration Research*,* 19 *(*2*),* 237–245; 37488872 10.4103/1673-5374.379041 PMC10503611

[3] Ajmeera, D., Ajumeera, R. (2023). Drug repurposing: A novel strategy to target cancer stem cells and therapeutic resistance. Genes and Diseases*,* 11 *(*1*),* 148–175; 37588226 10.1016/j.gendis.2022.12.013 PMC10425757

[4] Brouwer, I., de Kort, M. A. C., Lenstra, T. L. (2024). Measuring transcription dynamics of individual genes inside living cells. Methods in Molecular Biology*,* 2694*,* 235–265; 37824008 10.1007/978-1-0716-3377-9_12

[5] Petrella, F., Toffalorio, F., Brizzola, S., De Pas, T. M., Rizzo, S. et al. (2014). Stem cell transplantation effectively occludes bronchopleural fistula in an animal model. Annals of Thoracic Surgery*,* 97*,* 480–483; 24370201 10.1016/j.athoracsur.2013.10.032

[6] Petrella, F., Acocella, F., Barberis, M., Bellomi, M., Brizzola, S. et al. (2015). Airway fistula closure after stem-cell infusion. New England Journal of Medicine*,* 372*,* 96–97; 25551543 10.1056/NEJMc1411374

[7] Coccè, V., Franzè, S., Brini, A. T., Giannì, A. B., Pascucci, L. et al. (2019). *In vitro* anticancer activity of extracellular vesicles (EVs) secreted by gingival mesenchymal stromal cells primed with paclitaxel. Pharmaceutics*,* 11 *(*2*),* 61. 30717104 10.3390/pharmaceutics11020061 PMC6409699

[8] Romberg, S. I., Kreis, N. N., Friemel, A., Roth, S., Souto, A. S. et al. (2022). Human placental mesenchymal stromal cells are ciliated and their ciliation is compromised in preeclampsia. BMC Medicine*,* 20 *(*1*),* 35; 35081949 10.1186/s12916-021-02203-1 PMC8793243

[9] Zhang, H., Cai, W., Xu, D., Liu, J., Zhao, Q. et al. (2023). Effects of mesenchymal stem cells on Treg cells in rats with colitis. Clinical Experimental Immunology*,* 214 *(*3*),* 296–303. 10.1093/cei/uxad072; 37417713 PMC10719214

[10] Joshi, J. M., Muttigi, M. S., Upadhya, R., Seetharam, R. N. (2023). An overview of the current advances in the treatment of inflammatory diseases using mesenchymal stromal cell secretome. Immunopharmacology and Immunotoxicology*,* 45 *(*4*),* 497–507; 36786742 10.1080/08923973.2023.2180388

[11] Mahjoor, M., Fakouri, A., Farokhi, S., Nazari, H., Afkhami, H. et al. (2023). Regenerative potential of mesenchymal stromal cells in wound healing: Unveiling the influence of normoxic and hypoxic environments. Frontiers in Cell and Developmental Biology*,* 11*,* 1245872; 37900276 10.3389/fcell.2023.1245872 PMC10603205

[12] Cheng, J., Sun, Y., Ma, Y., Ao, Y., Hu, X. et al. (2022). Engineering of MSC-derived exosomes: A promising cell-free therapy for osteoarthritis. Membranes*,* 12 *(*8*),* 739; 36005656 10.3390/membranes12080739 PMC9413347

[13] Fang, X., Lan, H., Jin, K., Qian, J. (2023). Pancreatic cancer and exosomes: Role in progression, diagnosis, monitoring, and treatment. Frontiers in Oncology*,* 13*,* 1149551; 37287924 10.3389/fonc.2023.1149551 PMC10242099

[14] Coccè, V., Bonomi, A., Cavicchini, L., Sisto, F., Giannì, A. et al. (2020). Paclitaxel priming of TRAIL expressing mesenchymal stromal cells (MSCs-TRAIL) increases antitumor efficacy of their secretome. Current Cancer Drug Targets*,* 21 *(*3*),* 213–222. 10.2174/1568009620666201116112153; 33200709

[15] Fan, D., Cao, Y., Cao, M., Wang, Y., Cao, Y. et al. (2023). Nanomedicine in cancer therapy. Signal Transduction and Target Therapy*,* 8 *(*1*),* 293; 37544972 10.1038/s41392-023-01536-y PMC10404590

[16] Sababathy, M., Ramanathan, G., Tan, S. C. (2022). Targeted delivery of gold nanoparticles by neural stem cells to glioblastoma for enhanced radiation therapy: A review. AIMS Neuroscience*,* 9 *(*3*),* 303–319; 36329899 10.3934/Neuroscience.2022017 PMC9581732

[17] Liu, W. S., Wu, L. L., Chen, C. M., Zheng, H., Gao, J. et al. (2023). Lipid-hybrid cell-derived biomimetic functional materials: A state-of-the-art multifunctional weapon against tumors. Materials Today Bio*,* 22*,* 100751; 37636983 10.1016/j.mtbio.2023.100751 PMC10448342

[18] Nethi, S. K., Li, X., Bhatnagar, S., Prabha, S. (2023). Enhancing anticancer efficacy of chemotherapeutics using targeting ligand-functionalized synthetic antigen receptor-mesenchymal stem cells. Pharmaceutics*,* 15 *(*6*),* 1742; 37376189 10.3390/pharmaceutics15061742 PMC10304812

[19] Zhang, Y., Deng, H., Hu, Y., Pan, C., Wu, G. (2019). Adipose-derived mesenchymal stem cells stereotactic transplantation alleviate brain edema from intracerebral hemorrhage. Journal of Cellular Biochemestry*,* 120 *(*9*),* 14372–14382; 30963640 10.1002/jcb.28693

[20] Wang, C., Cao, J., Duan, S., Xu, R., Yu, H. et al. (2020). Effect of microRNA-126a-3p on bone marrow mesenchymal stem cells repairing blood-brain barrier and nerve injury after intracerebral hemorrhage. Journal of Stroke and Cerebrovascular Diseases*,* 29 *(*5*),* 104748; 32160957 10.1016/j.jstrokecerebrovasdis.2020.104748

[21] Chen, L., Zhang, N., Huang, Y., Zhang, Q., Fang, Y. et al. (2023). Multiple dimensions of using mesenchymal stem cells for treating liver diseases: From bench to beside. Stem Cell Reviews and Reports*,* 19 *(*7*),* 2192–2224; 37498509 10.1007/s12015-023-10583-5

[22] Salagean, A., Nechifor-Boila, A., Bajwa, N., Pastorello, Y., Slevin, M. (2023). Micro-fragmented adipose tissue as a natural scaffold for targeted drug delivery in brain cancer. International Journal of Molecular Sciences*,* 24 *(*14*),* 11530; 37511289 10.3390/ijms241411530 PMC10380718

[23] Ding, Y., Wang, C., Sun, Z., Wu, Y., You, W. et al. (2021). Mesenchymal stem cells engineered by nonviral vectors: A powerful tool in cancer gene therapy. Pharmaceutics*,* 13 *(*6*),* 913; 34205513 10.3390/pharmaceutics13060913 PMC8235299

[24] Tesiye, M. R., Gol, M., Fadardi, M. R., Kani, S. N. M., Costa, A. M. et al. (2022). Therapeutic potential of mesenchymal stem cells in the treatment of epilepsy and their interaction with antiseizure medications. Cells*,* 11 *(*24*),* 4129; 36552892 10.3390/cells11244129 PMC9777461

[25] Xiao, Y., Xu, R. H., Dai, Y. (2023). Nanoghosts: Harnessing mesenchymal stem cell membrane for construction of drug delivery platforms via optimized biomimetics. Small*,* e2304824. 10.1002/smll.202304824; 37653618

[26] Rachakatla, R. S., Balivada, S., Seo, G. M., Myers, C. B., Wang, H. et al. (2010). Attenuation of mouse melanoma by A/C magnetic field after delivery of bi-magnetic nanoparticles by neural progenitor cells. ACS Nano*,* 4*,* 7093–7104; 21058696 10.1021/nn100870z PMC3011034

[27] Raimondi, S., Maisonneuve, P., Lowenfels, A. B. (2009). Epidemiology of pancreatic cancer: An overview. Nature Reviews Gastroenterology & Hepatology*,* 6*,* 699–708. 19806144 10.1038/nrgastro.2009.177

[28] Li, D., Xie, K., Wolff, R., Abbruzzese, J. L. (2004). Pancreatic cancer. Lancet*,* 363*,* 1049–1057; 15051286 10.1016/S0140-6736(04)15841-8

[29] Hines, O. J. R., Howard, A. (2008). Pancreatic surgery. Current Opinions in Gastroenterology*,* 24*,* 603–611; 19122502 10.1097/MOG.0b013e32830b112e

[30] Wee, C. W., Kim, E., Kim, T. M., Park, C. K., Kim, J. W. et al. (2017). Impact of interim progression during the surgery-to-radiotherapy interval and its predictors in glioblastoma treated with temozolomide-based radiochemotherapy. Journal of Neurooncology*,* 134*,* 169–175; 28547592 10.1007/s11060-017-2505-x

[31] Zhao, J., Zhang, Z., Cui, Q., Zhao, L., Hu, Y. et al. (2020). Human adipose-derived mesenchymal stem cells inhibit proliferation and induce apoptosis of human gastric cancer HGC-27 cells. 3Biotech*,* 10 *(*3*),* 129. 10.1007/s13205-020-2090-0 PMC7035394 32154042

[32] Serhal, R., Saliba, N., Hilal, G., Moussa, M., Hassan, G. S. et al. (2019). Effect of adipose-derived mesenchymal stem cells on hepatocellular carcinoma: *In vitro* inhibition of carcinogenesis. World Journal of Gastroenterology*,* 25 *(*5*),* 567–583; 30774272 10.3748/wjg.v25.i5.567 PMC6371009

[33] Zakaria, N., Yahaya, B. H. (2020). Adipose-derived mesenchymal stem cells promote growth and migration of lung adenocarcinoma cancer cells. Advances in Experimental Medicine and Biology*,* 1292*,* 83–95; 31916234 10.1007/5584_2019_464

[34] Wu, S., Wang, Y., Yuan, Z., Wang, S., Du, H. et al. (2019). Human adipose‐derived mesenchymal stem cells promote breast cancer MCF7 cell epithelial‐mesenchymal transition by cross interacting with the TGF‐β/Smad and PI3K/AKT signaling pathways. Molecular Medicine Reports*,* 19 *(*1*),* 177–186; 30483746 10.3892/mmr.2018.9664 PMC6297785

[35] Pessina, A., Leonetti, C., Artuso, S., Benetti, A., Dessy, E. et al. (2015). Drug-releasing mesenchymal cells strongly suppress B16 lung metastasis in a syngeneic murine model. Journal of Experimetal & Clinical Cancer Research*,* 34*,* 82. 10.1186/s13046-015-0200-3 PMC4534150 26264809

[36] Bonomi, A., Steimberg, N., Benetti, A., Berenzi, A., Alessandri, G. et al. (2023). Paclitaxel-releasing mesenchymal stromal cells inhibit the growth of multiple myeloma cells in a dynamic 3D culture system. Hematology Oncology Small*,* 19 *(*40*),* e2301748. 10.1002/smll.202301748 27283119

[37] Cao, Z., Li, P., Li, Y., Zhang, M., Hao, M. et al. (2023). Encapsulation of nano-bortezomib in apoptotic stem cell-derived vesicles for the treatment of multiple myeloma. Small*,* 19 *(*40*),* e2301748; 37282762 10.1002/smll.202301748

[38] Wu, Y. W., Lee, D. Y., Lu, Y. L., Delila, L., Nebie, O. et al. (2023). Platelet extracellular vesicles are efficient delivery vehicles of doxorubicin, an anti-cancer drug: Preparation and *in vitro* characterization. Platelets*,* 34 *(*1*),* 2237134; 37580876 10.1080/09537104.2023.2237134

[39] Azizsoltani, A., Hatami, B., Zali, M. R., Mahdavi, V., Baghaei, K. et al. (2023). Acid-loaded exosomes attenuate liver fibrosis through dual targeting of the FXR signaling pathway and ECM remodeling. Biomedical Pharmacotherapy*,* 168*,* 115777. 10.1016/j.biopha.2023.115777 37913732

[40] Azarifar, Z., Amini, R., Tanzadehpanah, H., Afshar, S., Najafi, R. (2023). *In vitro* co-delivery of 5-fluorouracil and all-trans retinoic acid by PEGylated liposomes for colorectal cancer treatment. Molecular Biology Reports*,* 50 *(*12*),* 10047–10059. 10.1007/s11033-023-08888-2; 37902908

[41] Bharathi, R., Harini, G., Sankaranarayanan, A., Shanmugavadivu, A., Vairamani, M. et al. (2023). Nuciferine-loaded chitosan hydrogel-integrated 3D-printed polylactic acid scaffolds for bone tissue engineering: A combinatorial approach. International Journal of Biological Macromolecules*,* 253 *(*7*),* 127492; 37858655 10.1016/j.ijbiomac.2023.127492

[42] Sayyed, A. A., Gondaliya, P., Yan, I. K., Carrington, J., Driscoll, J. et al. (2023). Engineering cell-derived nanovesicles for targeted immunomodulation. Nanomaterials*,* 13 *(*20*),* 2751; 37887902 10.3390/nano13202751 PMC10609599

[43] He, X., Yao, W., Zhu, J. D., Jin, X., Liu, X. Y. et al. (2023). Potent antitumor efficacy of human dental pulp stem cells armed with YSCH-01 oncolytic adenovirus. Journal of Translational Medicine*,* 21 *(*1*),* 688; 37789452 10.1186/s12967-023-04539-z PMC10546667

